# Entropy Production and Irreversibility in the Linearized Stochastic Amari Neural Model

**DOI:** 10.3390/e27111104

**Published:** 2025-10-25

**Authors:** Dario Lucente, Giacomo Gradenigo, Luca Salasnich

**Affiliations:** 1 Department of Mathematics & Physics, University of Campania “Luigi Vanvitelli”, Viale Lincoln 5, 81100 Caserta, Italy; dario.lucente@unicampania.it; 2 Gran Sasso Science Institute (GSSI), Viale F. Crispi 7, 67100 L’Aquila, Italy; giacomo.gradenigo@gssi.it; 3 INFN-LNGS, Via Giovanni Acitelli, 22, 67100 L’Aquila, Italy; 4 Dipartimento di Fisica e Astronomia “Galileo Galilei”, Università di Padova, Via Marzolo 8, 35131 Padova, Italy; 5 INFN, Sezione di Padova, via Marzolo 8, 35131 Padova, Italy

**Keywords:** entropy production, neural dynamics, stochastic processes

## Abstract

One among the most intriguing results coming from the application of statistical mechanics to the study of the brain is the understanding that it, as a dynamical system, is inherently out of equilibrium. In the realm of non-equilibrium statistical mechanics and stochastic processes, the standard observable computed to determine whether a system is at equilibrium or not is the entropy produced along the dynamics. For this reason, we present here a detailed calculation of the entropy production in the Amari model, a coarse-grained model of the brain neural network, consisting of an integro-differential equation for the neural activity field, when stochasticity is added to the original dynamics. Since the way to add stochasticity is always to some extent arbitrary, particularly for coarse-grained models, there is no general prescription to do so. We precisely investigate the interplay between noise properties and the original model features, discussing in which cases the stationary state is in thermal equilibrium and which cases it is out of equilibrium, providing explicit and simple formulae. Following the derivation for the particular case considered, we also show how the entropy production rate is related to the variation in time of the Shannon entropy of the system.

## 1. Neural Dynamics Irreversibility and Statistical Mechanics

One possible description of large-scale brain activity is the one given by coarse-grained models based on the dynamics of neural fields. In such models, the average activity of a large neuronal population at a given position is encoded in the specific form of the integro-differential equations which govern the field dynamics [1]. Such a description is envisaged for understanding the behavior of a large number of interacting units without including all possible microscopic details of the real dynamics. Since the pioneering contribution of Wilson and Cowan [2] and Amari [3,4], tools from both equilibrium and non-equilibrium statistical mechanics, with particular reference to the emergence of critical behaviors [1,5,6,7,8,9], have been extensively used for characterizing neural activity. In particular, the necessity to consider *non-equilibrium* statistical mechanics has been motivated by the evidence that brain activity exhibits features of an intrinsically irreversible process [10]. An issue which therefore stays at the forefront of the interplay between theoretical physics and neuroscience is how to use the tools of non-equilibrium statistical mechanics to ascertain the irreversible nature of brain dynamics from data. To this end, among other areas, the study of fluctuation–dissipation relationships, namely how the internal correlations of a given system are connected to its response to an external perturbation, has been the most widely considered. The crucial feature of fluctuation–dissipation relationships is that they take a different form depending on whether the nature of system dynamics is irreversible or not. These relationships have therefore been considered to study the functional relationship between the evoked response to external stimuli and steady-state correlations in brain activity [11,12], revealing its irreversible nature [13]. The main limitation of the fluctuation–dissipation approach to the study of irreversibility dynamics in brain activity is the necessity to perform response experiments: the possibility to ascertain the nature of its dynamics by simply monitoring specific indicators without the intervention of external stimuli is therefore appealing. For this purpose, it is quite useful to study how fluctuations in relevant observables related to brain activity are correlated in time, particularly functions of the kind $C(t^{\prime \prime },t^{\prime })$, for which we do not need to specify either the specific observable or the structure of the function, but we just need to know that $t^{\prime }$ is the time of the first detection of the signal and $t^{\prime \prime }$ is the time of the second detection of the signal, $t^{\prime \prime }>t^{\prime }$. The role of $C(t^{\prime \prime },t^{\prime })$ is then to tell us how the fluctuations at $t^{\prime \prime }$ are correlated to those at $t^{\prime }$. By definition, the system obtains an equilibrium/reversible dynamics when the time-ordered pattern of fluctuations is statistically equivalent when the same sequence is considered from $t^{\prime }$ to $t^{\prime \prime }$ or from $t^{\prime \prime }$ to $t^{\prime }$. In this situation, the dynamics of the system are said to experience *time reversal* symmetry, in which case the correlation function $C(t^{\prime \prime },t^{\prime })$ also has the same symmetry, i.e., $C\left(t^{\prime \prime },t^{\prime }\right)=C\left(t^{\prime },t^{\prime \prime }\right)$. It is then sufficient to spot a single observable for which the above symmetry is broken, i.e., $C\left(t^{\prime \prime },t^{\prime }\right)\neq C\left(t^{\prime },t^{\prime \prime }\right)$, to say that the time reversal symmetry is broken, namely the system is *irreversible*. Unfortunately the behavior of the time-correlation functions strongly depend on the choice of the observables and on their specific functional form, which must often be chosen case by case. While the use of a time-correlation function presents a clear advantage, namely that one does need a specific model for the system’s microscopic dynamics, it also has a major limitation: the fact that it is not clear in all cases a priori what the correct observable to choose is for the correlation functions [14]. On the contrary, an *objective* and universal indicator of the lack of thermal equilibrium is the so-called *entropy production rate*, which is zero for systems at thermal equilibrium and positive for systems with irreversible dynamics, and it can be measured directly from *“unperturbed”* dynamics, namely from the spontaneous dynamical evolution of the system, without applying external stimuli. The only limitation of entropy production is that in order to obtain a reliable estimate of it, one needs to introduce an appropriate theoretical model of the system: it cannot be computed solely from data without any assumptions on the underlying model [14]. It is for this reason that, if one wishes to study the irreversibility, and hence entropy production, from brain data in connection to the Amari model, the first task is to study the explicit formula for entropy production which can be obtained for the case at hand. In particular, since the original equations of the model are deterministic, a necessary technical step in order to use the standard formalism to compute entropy production, without considering the formalism proper for deterministic systems [15], is to randomize the dynamics. The task of the present discussion is precisely to show how different methods of dynamic randomization are connected to the intrinsic properties of the models yielding different results for entropy production.

This paper is structured as follows: In Section 2, we recall from the realm of non-equilibrium statistical mechanics and stochastic thermodynamics the modern definition of entropy production in stochastic systems, first provided in a famous paper by Lebowitz and Spohn [16], also mentioning that it is related to the Shannon entropy of the system of interest. The purpose of this section is also to motivate the choice of entropy production rather than observables to characterize the degree of irreversibility in the system. Since the tools introduced in Section 2 apply specifically to stochastic systems, in Section 3, we introduce a linearized version of the Amari equation, proposing a standard protocol to randomize it and commenting on which version is the simplest choice in terms of model properties, which leads to an equilibrium state with reversible dynamics, where the entropy production is zero. Then in Section 4, we present our first original result: the complete calculation of entropy production as an indicator of irreversibility in the Lebowitz–Spohn approach, where the probability of trajectories forward in time is compared with the probability of trajectories backward in time. The resulting expression clarifies the peculiar interplay between the Amari model’s properties and the properties of the superimposed stochasticity in determining the reversible or irreversible nature of the dynamics. Section 5 is then dedicated to showing how the same expression obtained in Section 4 from the Lebowitz–Spohn formalism can be obtained by different means by studying the rate of variation in time of the Shannon entropy, another concept typical of stochastic thermodynamics. The peculiarity of this section is the discussion in terms of a functional formalism for both the Shannon entropy and the Fokker–Planck equation governing the stochastic dynamics of the neural activity field, which is not so common in the literature. Furthermore, by comparing the Lebowitz–Spohn entropy production with the variation in Shannon entropy, this section is useful for gaining better physical insights into the object of study. Section 6 is then dedicated to showing how a simple explicit expression of entropy production can be computed when assuming translational invariance in the space of coordinates for the original equation, making transparent the role played by the symmetry properties of the linearized Amari equation force. Finally in Section 7, we present our conclusions.

## 2. Entropy Production in Non-Equilibrium Statistical Mechanics

As noted in the previous section, an important concept that neuroscience, in particular the study of brain activity, has borrowed from non-equilibrium statistical mechanics is entropy production [17,18], a measurable quantity which characterizes the degree of irreversibility of brain dynamics. For instance, entropy production has been related to the hierarchical and asymmetrical structure of brain connections [19,20,21], with techniques also being successfully applied to the analysis of experimental signals [10,11]. It has also been shown that different estimates of entropy production from empirical datasets reveal a correlation to the consciousness level and the activity recorded in subjects performing different tasks [10,13,19,22]. Moreover, an interesting decomposition of entropy production in oscillatory modes has been applied to study how the brain’s rhythms contribute to dissipation and information processing [23]. Let us therefore define and elucidate the meaning of entropy production. The fact that entropy, which is a state function of a macroscopic system, grows along an irreversible transformation is a well-known fact from the 19th century. An account of how to quantitatively relate the entropy constantly produced along the irreversible dynamics of a stationary non-equilibrium state to macroscopic transport coefficients can be found in a historical book by De Groot and Mazur [24], which presents an overview of the development of this subject in the second half of the 20th century. Determining how the entropy produced macroscopically along stationary irreversible dynamics can be related to the probability of microscopic trajectories is a much more recent achievement. The standard way to compute entropy production in all stochastic processes was settled in the seminal paper by Lebowitz and Spohn in 1999 [16]. This work was proposed to extend the definition of Gallavotti–Cohen symmetry for the probability of stationary non-equilibrium fluctuations, initially conceived for deterministic dissipative systems, to systems which can be characterized in terms of a Markov chain. While in deterministic systems, the entropy produced macroscopically can be related, within the framework of a refined mathematical analysis, to the rate of contraction of phase space [15], the work of Lebowitz and Spohn was the first to provide an explicit formula to relate the same quantity to the probability of system trajectories. And, let us add, it is precisely the overwhelming simplicity of computing entropy production in the presence of stochasticity that motivated our present work, where we introduce the stochastic version of the Amari equation and explicitly compute the related entropy production rate. According to the Lebowitz–Spohn formula, if one considers the time interval [0,t] and denotes the sequence of configurations visited by the system in this interval as $\Omega_{0}^{t}$, which is usually denoted as *forward* trajectory, and the reversed sequence of configurations as ${\bar{\Omega }}_{0}^{t}$, which is usually denoted as *backward* trajectory, the entropy produced along the time span [0,t] is [14,25]

(1) $$\begin{array}{c} \Sigma \left(t\right)=\log\left(\frac{P[\Omega_{0}^{t}]}{P[{\bar{\Omega }}_{0}^{t}]}\right) \end{array}$$

One of the most interesting properties of entropy production is that in general, i.e., for systems with finite-term memory, it can be shown that Σ(t), which depends on the stochasticity of the dynamics, has a self-averaging property [26]; namely, in the case of a large system size, its probability distribution naturally concentrates around the average, as shown in the following:

(2) $$\begin{array}{c} \lim_{t\to \infty }\frac{\Sigma (t)}{t}=\lim_{t\to \infty }\frac{\langle \Sigma (t)\rangle }{t}, \end{array}$$

where the average is taken with respect to the probability of forward trajectories, and we divide by *t* in order to obtain a finite quantity, since Σ(t) is by definition extensive in *t*. Therefore, since after a long time, we have Σ(t)≈〈Σ(t)〉, the properties of the typical value of entropy production for t≫1 are those of the mean, which reads as

(3) $$\begin{array}{c} \langle \Sigma \left(t\right)\rangle =\int D\Omega_{0}^{t}\;P\left(\Omega_{0}^{t}\right)\;\log\left(\frac{P[\Omega_{0}^{t}]}{P[{\bar{\Omega }}_{0}^{t}]}\right). \end{array}$$

What is nice about the expression in Equation (3) is that it explicitly takes the form of Kullback–Leibler divergence. This is particularly interesting for motivating the choice of entropy production rather than correlation functions in order to assess the degree of irreversibility of the dynamics. As the distance between two probability distributions, Kullback–Leibler divergence does not depend on the choice of variables, as long as we have the probabilities $P(\Omega_{0}^{t})$ and $P({\bar{\Omega }}_{0}^{t})$. This gives entropy production a great degree of universality: it does not depend on the variables we choose to represent the system trajectories, as long as we are able to determine with precision their probability. In addition, if some degrees of freedom are integrated out, the Kullback–Leibler divergence cannot increase, meaning that the entropy production rate decreases under coarse-graining [14]. On the other hand, the “limit” of this approach is that we need precise mathematical modeling for the dynamics of our system; otherwise it is quite unlikely that we will obtain correct probability distributions. The general manipulations conducted in the literature, starting from the definition of entropy production in Equation (3), are usually aimed at writing the stationary entropy production rate in terms of the appropriate combination of correlation functions, a task which is accomplished by computing, in any specific case, the quantity

(4) $$\begin{array}{c} \sigma =\lim_{t\to \infty }\frac{\Sigma (t)}{t}\;\;=\;\;\mathrm{stationary}\;\mathrm{correlators}. \end{array}$$

The purpose of the work presented here is precisely to illustrate how by adding stochasticity to the originally deterministic Amari model, it is possible to exploit the entropy production formulae that are widespread in the contemporary literature [15,16,27,28]. In addition to this, we will show how the same expression for the entropy production rate can be obtained, partly by using known formulae [25,27,29] and also starting from the Shannon entropy formula from stochastic thermodynamics. This alternative derivation of the same formulae will clarify the whole picture, connecting entropy production with the time variation in the stationary entropy of the system. Let us assume that all variables needed to specify the state of the system are indicated by *X* and that their time-dependent probability distribution P(X,t) is known. In this case, the Shannon entropy of the system is

(5) $$\begin{array}{c} S_{\mathrm{sys}}\left(t\right)=-\int DX\;P\left(X,t\right)\;\log\left[P\left(X,t\right)\right], \end{array}$$

where we use the symbol DX to denote functional integration over a possibly multidimensional space. By using simple formal manipulations, and also the Fokker–Planck equation of which P(X,t) is a solution, this is a standard procedure to show that the time derivative of the system entropy, defined as the Shannon entropy in Equation (5), splits into the difference in two contributions, which are usually interpreted, respectively, as the entropy of the universe, $S_{\mathrm{tot}}$, and the entropy of the reservoir coupled to the system, $S_{\mathrm{res}}$:

(6) $$\begin{array}{c} {\dot{S}}_{\mathrm{sys}}\left(t\right)={\dot{S}}_{\mathrm{tot}}\left(t\right)-{\dot{S}}_{\mathrm{res}}\left(t\right). \end{array}$$

In a stationary state, the probability distribution P(X,t) is stationary, so the entropy of the system is also constant, and ${\dot{S}}_{\mathrm{sys}}\left(t\right)=0$. In this case, the rate of increase in the entropy of the universe and the entropy of the reservoir are identical, ${\dot{S}}_{\mathrm{tot}}\left(t\right)={\dot{S}}_{\mathrm{res}}\left(t\right)$, and we will show that they also correspond to the system’s entropy production rate as computed from a Lebowitz–Spohn-like functional. So far, we have kept the discussion general in order to present all the aspects of entropy production that are non-specific to a particular model. The next section will be devoted to the presentation of the Amari model, which we chose as a benchmark to study entropy production in a model for brain dynamics.

## 3. Linearized Stochastic Amari Model: Equilibrium Properties

In the previous section, we introduced the concept of the entropy production rate as an indicator for the degree of irreversibility in system dynamics. We also mentioned how, due to the Lebowitz–Spohn generalization of the Gallavotti–Cohen results, the calculation of entropy production is much easier in a system with stochasticity. It is for this reason that, elaborating on its original deterministic version, we added stochasticity to the Amari model for neural dynamics. Before presenting the calculation of entropy production in this model, let us introduce the model and explain the motivation behind this choice.

The stochastic Amari model is an integro-differential stochastic equation which models the neural activity of the brain by means of a local activation field u(x,t) defined in a spatio-temporal domain, $x\in \Omega \subset R^{d}$ and t∈R (we also assume Ω to be a boundary-free domain):

(7) $$\frac{\partial }{\partial t}u\left(x,t\right)=-\frac{1}{\tau }u\left(x,t\right)+\frac{1}{\tau }\int_{\Omega }w\left(x,y\right)\;f\left[u\left(y,t\right)\right]\;dy+\xi \left(x,t\right)\;,$$

where τ is the relaxation time, and the two-point function w(x,y), which models the transmission of impulses from one region to another of the brain, is usually known as the synaptic weight. In Equation (7), f[u(x,t)] then appears, the activation function, which is expected to be saturated to a constant value when the local field exceeds a certain threshold $u^{*}$. Usually the activation function is modeled as a sigmoid:

(8) $$f\left[u\right]=\frac{1}{e^{\beta (u^{*}-u)}+1}\;$$

with gain β>0 and threshold $u^{*}>0$. For the present discussion, we will consider ξ(x,t) to be a Gaussian field delta-correlated in time, i.e., white noise. Besides the correlation in the time domain, the noise field is also characterized by its spatial covariance:

(9) $$\begin{array}{c} \langle \xi \left(x,t\right)\xi \left(y,t^{\prime }\right)\rangle =\gamma \left(x,y\right)\;\delta \left(t-t^{\prime }\right), \end{array}$$

where, in order to obtain a consistent definition of noise, its covariance γ(x,y) must be a symmetric function of its coordinates:

(10) $$\begin{array}{c} \gamma (x,y)=\gamma (y,x). \end{array}$$

For simplicity, here, we only consider the case of additive noise. Thus, Equation (7) does not suffer from a different physical interpretation resulting from different discretization schemes [30]. This choice allows us to focus on the role of synaptic couplings and noise correlations in sustaining irreversible stationary states without facing all the difficulties encountered when multiplicative noise, with possibly time-varying power-law behavior, is taken into account [31]. We chose a stochastic version of the Amari neural field equation because it provides a minimal, spatially continuous mean-field description that directly links macroscopic cortical activity to physiologically interpretable ingredients: the synaptic coupling kernel w(x,y) encodes anatomical connectivity, the non-linear gain function f[u(x,t)] represents neuronal input–output properties, and the explicit temporal decay modeled by the term $-\tau^{-1}u\left(x,t\right)$ represents membrane/leak dynamics. The deterministic Amari field u(x,t) was originally introduced as a canonical model for pattern formation and spatially structured activity in the cortex (bumps, waves, spatial patterns), as discussed in Chap. 1 of Ref. [32]. The addition of stochastic forcing to this framework is not only a technical trick to allow for a much easier calculation of entropy production but also a natural and well-studied extension because real cortical tissue is subject to numerous sources of variability (synaptic noise, finite-size effects, background inputs) whose principal effects are captured at the continuum level by noise terms (see Chap. 9 of Ref. [32]). In addition to this, stochastic neural field equations have been used to analyze phenomena such as the noise-induced wandering of stationary bumps, diffusion of wave positions, variability in stimulus tuning, and noise-driven bifurcations of spatial patterns—phenomena directly relevant to working memory, perceptual switching, and cortical variability observed in experiments [4,32,33]. From a mathematical perspective, the stochastic Amari model requires a rigorous probabilistic treatment (well-posedness, invariant measures, ergodicity) under reasonable assumptions on the synaptic kernel w(x,y) and noise correlations γ(x,y), which both justify the analytical approach taken here. This combination of physiological interpretability, a direct connection to experimentally observed noisy dynamics, and the mature mathematical literature motivated our choice of this model for randomization.

Since the Amari model is originally defined as a deterministic model, there is a priori some degree of arbitrariness in the choice of noise properties. In fact, whether the system attains a stationary state, which can be deemed as equilibrium, depends, as we are going to show, on the interplay between the properties of noise and those of the local force acting on the neural activity field. Here we start by showing which choice of noise yields equilibrium properties, leaving a more detailed study of the general case to the next section. As the first step in this direction, it is convenient to linearize the Amari equation around a stationary solution. One can therefore denote η(x,t) as the fluctuations around the homogeneous solution $u\left(x,t\right)=u_{0}$:

(11) $$\eta \left(x,t\right)=u\left(x,t\right)-u_{0}\;\;\;\;\left|\frac{u\left(x,t\right)-u_{0}}{u_{0}}\right|\ll 1.$$

We consider then the expansion to linear order in η(x,t) of the non-linear activation function f[u(x,t)]:

(12) $$f\left[u\left(x,t\right)\right]=f\left[u_{0}\right]+f^{\prime }\left[u_{0}\right]\eta \left(x,t\right)+...\;$$

The homogeneous solution $u_{0}$ is the one obtained by plugging $\eta \left(x,t\right)=u_{0}+u\left(x,t\right)$ into Equation (7), then expanding and solving for $u_{0}$ after having set $\dot{\eta }\left(x,t\right)=0$ and ξ(x,t)=0:

(13) $$0=-u_{0}+\tilde{w}\;f\left[u_{0}\right]\;$$

where

(14) $$\tilde{w}=\int_{\Omega }w\left(x,y\right)\;dy.$$

The claim that the integral on the right-hand side of Equation (14) yields a constant value $\tilde{w}$ independent from the coordinate x can be simply justified under the hypothesis that all points in the brain are equivalent from the perspective of the connectivity with the rest of the system, which is quite reasonable if one considers that all models for neural networks in the brain [34,35] are usually dense networks. For a given choice of $\tilde{w}$, the stationary homogeneous solution $u_{0}$ can be simply obtained from the following algebraic non-linear equation

(15) $$f\left[u_{0}\right]=\frac{u_{0}}{\tilde{w}}\;.$$

By plugging the expansion of Equation (11) into the stochastic Amari equation and truncating to the linear order, we get

(16) $$\frac{\partial }{\partial t}\eta \left(x,t\right)=-\frac{1}{\tau }\eta \left(x,t\right)+\frac{f^{\prime }\left[u_{0}\right]}{\tau }\int_{\Omega }w\left(x,y\right)\eta \left(y,t\right)dy+\xi \left(x,t\right)\;,$$

which is the stochastic linear Amari equation for the (small) fluctuations η(x,t) around the homogeneous solution $u_{0}$. The last equation can be rewritten in a more compact form as follows:

(17) $$\begin{array}{c} \dot{\eta }\left(x,t\right)=\Lambda \left[\eta \right]\left(x,t\right)+\xi \left(x,t\right), \end{array}$$

where Λ[η](x,t) is an integral operator, acting on the field η(x,t), defined by its kernel λ(x,y)

(18) $$\begin{array}{cc} \Lambda [\eta ](x,t) & =\int dy\;\lambda (x,y)\;\eta (y,t)\\ \lambda (x,y) & =-\frac{1}{\tau }\delta^{\left(d\right)}\left(x,y\right)+\frac{f^{\prime }\left[u_{0}\right]}{\tau }w\left(x,y\right) \end{array}$$

One of the goals of the following discussion is to study how the presence of a thermodynamic equilibrium depends on the properties of λ(x,y). Clearly, the operator Λ must be bounded and negative define to guarantee that Equation (17) is well-defined. For instance, a sufficient condition is that $\lambda \in L_{2}\left(\Omega \right)$, with $L_{2}\left(\Omega \right)$ being the space of the square-integrable function on Ω. At the same time, the noise correlation kernel γ must be positive definite, and for simplicity, we also assume it is invertible. It is well known that the synaptic weight w(x,y) is a symmetric function of the coordinates and Λ is a self-adjoint operator. Therefore, one can introduce the following potential:

(19) $$\begin{array}{c} U\left[\eta \right]=-\frac{1}{2}\int dx\;dy\;\eta \left(x\right)\lambda \left(x,y\right)\eta \left(y\right) \end{array}$$

and write the Langevin equation as

(20) $$\begin{array}{c} \dot{\eta }\left(x,t\right)=-\frac{\delta U[\eta ]}{\delta \eta (x,t)}+\xi \left(x,t\right). \end{array}$$

In this case it is sufficient to have a noise which is delta-correlated in both space and time, i.e., a delta-correlated spatial kernel

(21) $$\begin{array}{c} \gamma (x,y)=T\;\delta (x-y) \end{array}$$

in order to have a Boltzmann-like equilibrium distribution (here the Boltzmann constant $k_{B}$ is set equal to 1)

(22) $$\begin{array}{c} P_{\mathrm{eq}}\left[\eta \right]\propto \exp\left(-U[\eta ]/T\right). \end{array}$$

We will demonstrate in the following sections that there is a more general condition, also involving the properties of the noise kernel, which guarantees the presence of thermodynamic equilibrium.

## 4. Entropy Production from the Lebowitz–Spohn Formula

After the historical introduction on entropy production presented in Section 2, here we show how to compute it exactly in the stochastic Amari model. Let us just notice that, at variance with the original entropy production formula given in the Lebowitz–Spohn paper for Markov chains [16], where trajectories are written in terms of transition rates, the stochastic Amari model discussed here consists of continuous time stochastic processes, so we will need the functional Onsager–Machlup formalism to determine path probabilities [25,36,37]. First of all, we have to provide an explicit definition for trajectories, which were referred to only in an abstract way in Section 2. In the present discussion, the *forward* trajectory is represented by the collection of the field η(x,t) configurations, ordered chronologically

(23) $$\begin{array}{c} \Omega_{0}^{t}=\left\{\eta (x,s)\;|\;s\in [0,t]\right\}, \end{array}$$

while the symbol ${\bar{\Omega }}_{0}^{t}$ denotes the *backward* trajectory

(24) $$\begin{array}{c} {\bar{\Omega }}_{0}^{t}=\left\{\bar{\eta (x,s)}\;|\;s\in \left[0,t\right]\right\}. \end{array}$$

Here, the overline denotes the sequence of configurations defined as follows

(25) $$\begin{array}{cc} \bar{\eta (x,s)} & =\eta (x,t-s)\;\;\;s\;\in \;[0,t] \end{array}$$

(26) $$\begin{array}{cc} \frac{d}{ds}\bar{\eta (x,s)} & =-\frac{d}{dt}\eta \left(x,t-s\right)\;\;\;s\;\in \;\left[0,t\right]. \end{array}$$

The apparently ambiguous notation for the derivative in the right-hand term of Equation (26) must be interpreted as follows:

(27) $$\begin{array}{c} \frac{d}{dt}\eta \left(x,t-s\right)=\frac{d}{du}\eta \left(x,u\right){|}_{u=t-s}\;. \end{array}$$

The task is then to compute

(28) $$\begin{array}{c} \Sigma \left(t\right)=\log\left(\frac{P[\Omega_{0}^{t}]}{P[{\bar{\Omega }}_{0}^{t}]}\right), \end{array}$$

for which we need the expressions of $P[\Omega_{0}^{t}]$ and $P[{\bar{\Omega }}_{0}^{t}]$. According to the Onsager–Machlup formula, for which the derivation is standard in the case of additive noise, see, for instance, [25,29], the probability of forward and backward trajectories can be, respectively, written as follows:

(29) $$\begin{array}{cc} P[\Omega_{0}^{t}] & \propto \exp\left\{-\frac{1}{2}\int_{0}^{t}ds\int dx\;dy\left[\dot{\eta }\left(x,s\right)-\Lambda \left[\eta \right]\left(x,s\right)\right]\gamma {\left(x,y\right)}^{-1}\left[\dot{\eta }\left(y,s\right)-\Lambda \left[\eta \right]\left(y,s\right)\right]\right\}, \end{array}$$

(30) $$\begin{array}{cc} P[{\bar{\Omega }}_{0}^{t}] & \propto \exp\{-\frac{1}{2}\int_{0}^{t}ds\int dx\;dy\left[-\dot{\eta }\left(x,t-s\right)-\Lambda \left[\eta \right]\left(x,t-s\right)\right]\gamma {\left(x,y\right)}^{-1}\\ \; & \left[-\dot{\eta }\left(y,t-s\right)-\Lambda \left[\eta \right]\left(y,t-s\right)\right]\}. \end{array}$$

From the above formulae, a straightforward algebraic manipulation yields the total entropy production, which takes the form of an integral over the elapsed time:

(31) $$\begin{array}{cc} \Sigma (t) & =\int_{0}^{t}ds\int dx\;dy\;\left[\dot{\eta }\left(x,s\right)\gamma {\left(x,y\right)}^{-1}\Lambda \left[\eta \right]\left(y,s\right)+\Lambda \left[\eta \right]\left(x,t-s\right)\gamma {\left(x,y\right)}^{-1}\dot{\eta }\left(y,t-s\right)\right]\\ \; & =2\int_{0}^{t}ds\int dx\;dy\;\dot{\eta }\left(x,s\right)\gamma {\left(x,y\right)}^{-1}\Lambda \left[\eta \right]\left(y,s\right) \end{array}$$

where the last integral has to be performed with the Stratonovich prescription. This is not related to the discretization scheme adopted in the definition of the stochastic differential equation and can be derived by discretizing the dynamics in time intervals Δt, writing the path probability $P[\Omega_{0}^{t}]$, and formally taking the limit Δt→0, as originally conceived by Onsager [36,37]. This is the most general expression of the entropy production in the Amari model, which relies on no other assumption than the linearization of the equations of motion. No translational invariance in space or symmetry under the exchange of coordinates has been considered for the synaptic weight function w(x,y). It is then customary to consider the entropy production per unit time in the large time limit, i.e., σ(t)=Σ(t)/t, which reads as a time average and where, if the dynamics relax to a stationary state, it is possible to replace this average with the average over the stationary distribution, thus obtaining an expression in terms of stationary correlation functions:

(32) $$\begin{array}{cc} \sigma & =\lim_{t\to \infty }\frac{2}{t}\int dx\;dy\;dz\;\gamma {\left(x,y\right)}^{-1}\lambda \left(y,z\right)\dot{\eta }\left(x,s\right)\eta \left(z,s\right)\\ \; & =2\int dx\;dy\;dz\;\gamma {\left(x,y\right)}^{-1}\lambda \left(y,z\right)\langle \dot{\eta }\left(x\right)\eta \left(z\right)\rangle \end{array}$$

What is now crucial in the manipulation of Equation (32) is to assume the Stratonovich convention for the definition of stochastic integrals. By replacing $\dot{\eta }\left(x\right)$ with its expression according to the Langevin equation, Equation (17), one gets the following:

(33) $$\begin{array}{c} \sigma =2\int dx\;dy\;dz\;dz^{\prime }\;\gamma {\left(x,y\right)}^{-1}\lambda \left(y,z\right)\left[\lambda \left(x,z^{\prime }\right)\langle \eta \left(z^{\prime }\right)\eta \left(z\right)\rangle +\delta \left(x-z^{\prime }\right)\langle \xi \left(z^{\prime }\right)\eta \left(z\right)\rangle \right] \end{array}$$

The above formula for the entropy production rate σ consists of two terms: The first one involves only $c\left(z^{\prime },z\right)=\langle \eta \left(z^{\prime }\right)\eta \left(z\right)\rangle$, the equal-time two-point correlation function of the field. The other term depends on the *equal-time* correlation between the noise and the field, which is also nontrivial due to the Stratonovich prescription. Therefore, Equation (33) can be rewritten as

(34) $$\begin{array}{cc} \sigma & =2\int dx\;dy\;dz\;dz^{\prime }\;\gamma {\left(x,y\right)}^{-1}\lambda \left(y,z\right)\left[\lambda \left(x,z^{\prime }\right)c\left(z^{\prime },z\right)+\delta \left(x-z^{\prime }\right)\frac{1}{2}\gamma \left(z^{\prime },z\right)\right]\\ \; & =2\int dx\;dy\;dz\;dz^{\prime }\;\gamma {\left(x,y\right)}^{-1}\lambda \left(y,z\right)\lambda \left(x,z^{\prime }\right)c\left(z^{\prime },z\right)+\int dx\;dy\;dz\;\gamma {\left(x,y\right)}^{-1}\lambda \left(y,z\right)\gamma \left(x,z\right)\\ \; & =2\int dx\;dy\;dz\;dz^{\prime }\;\lambda^{T}\left(z^{\prime },x\right)\gamma {\left(x,y\right)}^{-1}\lambda \left(y,z\right)c\left(z,z^{\prime }\right)+\int dz\;\lambda \left(z,z\right)\;. \end{array}$$

Let us now rewrite the expression in Equation (34) in a more transparent form. In order to do so, let us first define the trace of a generic linear operator

(35) $$\begin{array}{c} K[\eta ]=\int dy\;k(x,y)\eta (y) \end{array}$$

as

(36) $$\begin{array}{c} \mathrm{Tr}[K]=\int dx\;k(x,x)\;. \end{array}$$

By also considering the Lyapunov equation, which is a fundamental equation that must be fulfilled in order to guarantee the existence of a stationary state in a linear system [38]

(37) $$\begin{array}{cc} \int dz\;\left[\lambda \left(x,z\right)c\left(z,y\right)+c\left(x,z\right)\lambda^{T}\left(z,y\right)\right] & =-\gamma (x,y)\;\\ \Lambda \circ C+{\left(\Lambda \circ C\right)}^{T} & =-\Gamma , \end{array}$$

it is possible to plug it into the expression of the entropy production rate in Equation (34), thus obtaining the following:

(38) $$\begin{array}{cc} \sigma & =2\int dx\;dy\;dz\;dz^{\prime }\;\lambda^{T}\left(z^{\prime },x\right)\gamma {\left(x,y\right)}^{-1}\lambda \left(y,z\right)c\left(z,z^{\prime }\right)+\int dz\;\lambda \left(z,z\right)\\ \; & =\mathrm{Tr}\left[2\Lambda^{T}\circ \Gamma^{-1}\circ \Lambda \circ C+\Lambda \right]\\ \; & =\mathrm{Tr}\left[\Lambda^{T}\circ \Gamma^{-1}\circ \Lambda \circ C\right]+\mathrm{Tr}\left[\Lambda^{T}\circ \Gamma^{-1}\circ \Lambda \circ C+\Lambda \right]\\ \; & =\mathrm{Tr}\left[\Lambda^{T}\circ \Gamma^{-1}\circ \Lambda \circ C\right]+\mathrm{Tr}\left[\Lambda^{T}\circ \Gamma^{-1}\circ \left(-C\circ \Lambda^{T}-\Gamma \right)+\Lambda \right]\\ \; & =\mathrm{Tr}\left[\Lambda^{T}\circ \Gamma^{-1}\circ \Lambda \circ C\right]-\mathrm{Tr}\left[\Lambda^{T}\circ \Gamma^{-1}\circ C\circ \Lambda^{T}\right]+\mathrm{Tr}\left[\Lambda -\Lambda^{T}\right]\\ \; & =\mathrm{Tr}\left[(\Lambda^{T}\circ \Gamma^{-1}-\Gamma^{-1}\circ \Lambda )\circ \Lambda \circ C\right] \end{array}$$

The above expression of entropy production in Equation (38) is the central result of our paper. We will show how it can be written in a more explicit and insightful form when translational invariance is also considered in Section 6. In order to clarify its derivation, let us remark that from the first to the last line of Equation (38), we took advantage of the properties of the trace and of several identities which we are going to list right now. First of all, from the second to the third line of Equation (38), we used the Lyapunov stability condition in the following form:

(39) $$\begin{array}{c} \Lambda \circ C+{\left(\Lambda \circ C\right)}^{T}=-\Gamma \;\;\;⟹\;\;\;\Lambda \circ C=-C\circ \Lambda^{T}-\Gamma \end{array}$$

In the passages of Equation (38), we also made use of the cyclic permutation of the trace; of the fact that for any matrix *A*, we have $\mathrm{Tr}\left[A\right]=\mathrm{Tr}\left[A^{T}\right]$; and of the fact that Γ and *C*, as well as their inverse, are symmetric. The above properties allowed us to write

(40) $$\begin{array}{cc} \mathrm{Tr}\left[\Lambda^{T}\circ \Gamma^{-1}\circ C\circ \Lambda^{T}\right] & =\mathrm{Tr}\left[{\left(\Lambda \circ C\circ \Gamma^{-1}\circ \Lambda \right)}^{T}\right]\\ \; & =\mathrm{Tr}\left[\Lambda \circ C\circ \Gamma^{-1}\circ \Lambda \right]=\mathrm{Tr}\left[\Gamma^{-1}\circ \Lambda \circ \Lambda \circ C\right], \end{array}$$

which, considering that the trace of an antisymmetric matrix is zero, $\mathrm{Tr}\left[\Lambda -\Lambda^{T}\right]=0$, led us to the last line of Equation (38). The expression in the last line of Equation (38) makes it clear that the condition

(41) $$\begin{array}{c} \Lambda^{T}\circ \Gamma^{-1}=\Gamma^{-1}\circ \Lambda \end{array}$$

is the one necessary to result in a zero entropy production rate and is therefore, as a consequence, the one necessary to have thermodynamic equilibrium. In order to rewrite the identity in Equation (41) in a more transparent way, let us multiply both terms on the left and right by the operator Γ, e.g., $\Lambda^{T}\circ \Gamma^{-1}\to \Gamma \circ \Lambda^{T}\circ \Gamma^{-1}\circ \Gamma$, which, by also recalling that $\Gamma =\Gamma^{T}$, yields

(42) $$\begin{array}{c} \Gamma^{T}\circ \Lambda^{T}=\Lambda \circ \Gamma , \end{array}$$

which is the general “equilibrium” condition we were seeking. A more transparent expression can be obtained by explicitly determining the action of the operators in Equation (41) on the activity fields, for instance, by writing Λ∘Γ[η](x) as

(43) $$\begin{array}{c} \Lambda \circ \Gamma [\eta ](x)=\int dz\;dy\;\lambda (x,z)\;\gamma (z,y)\;\eta (y). \end{array}$$

The identity in Equation (42) can be then more explicitly written as

(44) $$\begin{array}{cc} \left(\Lambda \circ \Gamma \right)\left[\eta \right]\left(x\right)-\left(\Gamma^{T}\circ \Lambda^{T}\right)\left[\eta \right]\left(x\right) & =\int dz\;dy\;\left[\lambda (x,z)\;\gamma (z,y)-\gamma (x,z)\lambda (z,y)\right]\;\eta \left(y\right)\\ \; & =\int \;dy\;\left[h(x,y)-h(y,x)\right]\;\eta \left(y\right)=0, \end{array}$$

where we introduced the function

(45) $$\begin{array}{c} h(x,y)=\int dz\;\lambda (x,z)\;\gamma (z,y). \end{array}$$

If one wishes to have thermodynamic equilibrium, the expression in the second line of Equation (44) must be zero independently from the choice of η(y), which in turn implies symmetry under the exchange of arguments for the function h(x,y). The need for this symmetry for h(x,y) tells us that the symmetry under the exchange of arguments of the synaptic kernel w(x,y) is not in itself a sufficient condition to guarantee thermal equilibrium; what is needed is the symmetry of h(x,y), which implies a nontrivial relationship between γ(x,y) and λ(x,y). It is only in the case of a white noise that is also delta-correlated across space, i.e., γ(x,y)=Tδ(x−y), that the symmetry of the synaptic kernel becomes sufficient to have equilibrium, since we have

(46) $$\begin{array}{c} h(x,y)=T\;\gamma (x,y). \end{array}$$

As shown in [14,39], in the finite-dimensional case, these relations are equivalent to the Onsager reciprocal relations [40]. As we approach the end of this section, a comment is in order: Since most people are more familiar with the concept of entropy rather than entropy production, it might be useful to show how the same expression of the entropy production obtained from the Lebowitz–Spohn approach connects to the variation in time of one of the possible definitions of entropy in our system, namely the Shannon entropy. This task is accomplished in the next subsection, showing how an expression identical to the one in Equation (38) can be obtained. We deem this sort of derivation quite useful to grasp a more profound intuition of what entropy production is, before moving to Section 6, where a more explicit expression is provided for the case of translational invariance.

## 5. Entropy Production from Shannon Entropy

As noted in the discussion above, it is quite instructive to explicitly derive the relationship between the entropy production defined within the Lebowitz0-Spohn approach, specifically conceived for stochastic systems, and the usual entropy considered in the canonical ensemble, namely the Shannon entropy, which, according to the prescription of stochastic thermodynamics [27,28,41], can be directly computed from the time-dependent functional probability distribution $P_{t}\left[\eta \right]$ of the field η(x,t) as

(47) $$S_{\mathrm{sys}}\left(t\right)=-\int D\eta \left(t\right)\;P_{t}\left[\eta \right]\log\left(P_{t}\left[\eta \right]\right)\;,$$

where the symbol $D\eta \left(t\right)=\prod_{x}d\eta \left(x,t\right)$ denotes functional integration over space at a given time *t*. In the case of thermodynamic equilibrium, when the probability distribution of the field η(x,t) does not depend on time and has the form of a Boltzmann weight, for instance, the one of Equation (22) in Section 3, the Shannon entropy turns out to be precisely the canonical entropy of the system, i.e., the one computed as S=β〈E〉−βF, where 〈E〉 and *F* are, respectively, the internal energy and the free energy at the inverse temperature β. Also, interpreting $P_{t}$ as the single-particle marginal distribution of an *N*-dimensional system, $S_{sys}$ coincides with that minus the H-function defined by Boltzmann [24]. But here we deal with a more general case. We will show how, by taking the time derivative of the entropy $S_{\mathrm{sys}}\left(t\right)$ defined above and making use of the Fokker–Planck equation, one ends up with terms among which it is possible to recognize precisely the same entropy production expression derived from the Lebowitz–Spohn approach, which we obtained in the previous section. This sort of derivation is quite general; see, for instance, [25,29]. Nevertheless, in most cases, the procedure is discussed in terms of the probability distribution of a random variable, not of a fluctuating field, so we deemed it helpful and not redundant to go through all steps of the derivation using a *functional* formalism, which is what is needed here, and considering the specific form taken by the Fokker–Planck equation in the presence of a functional formulation, which is not so common in the stochastic thermodynamic literature. As we have said, we are interested in the time derivative of $S_{\mathrm{sys}}\left(t\right)$, which is

(48) $$\begin{array}{cc} {\dot{S}}_{\mathrm{sys}}\left(t\right) & =-\int D\eta \left(t\right)\;\dot{P}\left[\eta \right]\left[\log\left(P_{t}\left[\eta \right]\right)+1\right]\;,\\ \; & =-\int D\eta \left(t\right)\;\dot{P}\left[\eta \right]\log P_{t}\left[\eta \right]. \end{array}$$

The second term added in the first line of Equation (48) was eliminated by exploiting the conservation of probability normalization

(49) $$\begin{array}{c} \int D\eta \left(t\right)\;\frac{d}{dt}P_{t}\left[\eta \right]=\frac{d}{dt}\left[\int D\eta \left(t\right)\;P_{t}\left[\eta \right]\right]=0. \end{array}$$

At this stage, we need to use the Fokker–Planck equation, which governs the dynamics of $P_{t}\left[\eta \left(x,t\right)\right]$, which we write here in the form of a functional continuity equation which relates the rate of change probability density to the negative divergence of a probability current (or flux), thus ensuring total probability conservation [42]:

(50) $$\begin{array}{cc} \frac{d}{dt}P_{t}\left[\eta \right] & =-\int dx\;\frac{\delta J[\eta ](x)}{\delta \eta (x,t)}\;, \end{array}$$

where the current is defined as

(51) $$\begin{array}{cc} J[\eta ](x) & =P_{t}\left[\eta \right]\int dy\left[\lambda \left(x,y\right)\eta \left(y,t\right)-\frac{1}{2}\gamma \left(x,y\right)\frac{\delta }{\delta \eta (y,t)}\log\left(P\left[\eta \right]\right)\right] \end{array}$$

Then, by inserting the time derivative of the probability distribution as written in Equation (50) into the expression of the Shannon entropy time derivative, Equation (48), one gets

(52) $$\begin{array}{c} {\dot{S}}_{\mathrm{sys}}\left(t\right)=\int D\eta \left(t\right)\left[\int dx\;\frac{\delta J[\eta ](x)}{\delta \eta (x,t)}\right]\log\left(P_{t}\left[\eta \right]\right)\;. \end{array}$$

By integrating the functional integral by parts, one obtains

(53) $$\begin{array}{c} {\dot{S}}_{\mathrm{sys}}\left(t\right)=-\int D\eta \;\int dx\;J\left[\eta \right]\left(x\right)\frac{\delta }{\delta \eta (x,t)}\log\left(P_{t}\left[\eta \right]\right)\;, \end{array}$$

where we assumed that the probability current and probability distribution vanish sufficiently rapidly at the boundaries so that the boundary contribution can be neglected. Let us notice that the above formulation is completely general; it does not depend on either the linearity of the model or any stationarity assumption. If one then takes into account the explicit expression of the current J[η](x) given in Equation (51) and explicitly substitutes $\frac{\delta }{\delta \eta (x,t)}\log\left(P_{t}\left[\eta \right]\right)$ with

(54) $$\frac{\delta }{\delta \eta (x,t)}\log\left(P_{t}\left[\eta \right]\right)=2\int dz\;\gamma^{-1}\left(x,z\right)\left\{\int dz^{\prime }\;\lambda \left(z,z^{\prime }\right)\eta \left(z^{\prime },t\right)-\frac{J[\eta ](z)}{P_{t}\left[\eta \right]}\right\}\;,$$

it is possible to show that the time derivative of the Shannon entropy written in Equation (53) splits into the sum of only two nontrivial contributions. These contributions, according to the standard conventions in stochastic thermodynamics [28,41], can be identified with respect to the total entropy $S_{\mathrm{tot}}\left(t\right)$ of the universe (system+reservoir) and the entropy of the reservoir $S_{\mathrm{res}}\left(t\right)$:

(55) $$\begin{array}{cc} {\dot{S}}_{\mathrm{sys}}\left(t\right) & ={\dot{S}}_{\mathrm{tot}}\left(t\right)-{\dot{S}}_{\mathrm{res}}\left(t\right) \end{array}$$

where we have

(56) $$\begin{array}{cc} \; & {\dot{S}}_{\mathrm{tot}}\left(t\right)=2\int D\left[\eta \right]\int dxdz\frac{J\left[\eta \right]\left(x\right)\gamma^{-1}\left(x,z\right)J\left[\eta \right]\left(z\right)}{P_{t}\left[\eta \right]}\;, \end{array}$$

(57) $$\begin{array}{cc} \; & {\dot{S}}_{res}\left(t\right)=2\int D\left[\eta \right]\int dxdzdz^{\prime }\;J\left[\eta \right]\left(x\right)\gamma^{-1}\left(x,z\right)\lambda \left(z,z^{\prime }\right)\eta \left(z^{\prime }\right)\;. \end{array}$$

The relationship between the rate of change in the Shannon entropy and the Lebowitz–Spohn approach emerges in the stationary state, where

(58) $$\begin{array}{c} \frac{d}{dt}P\left[\eta \right]=0\;\;\;⟹\;\;\;{\dot{S}}_{\mathrm{sys}}=0\;\;\;⟹\;\;\;{\dot{S}}_{\mathrm{tot}}={\dot{S}}_{\mathrm{res}} \end{array}$$

Physically, the last identity of Equation (58) means that in the stationary state, all of the entropy produced during the dynamics is dissipated into the environment/reservoir. The last step of the calculation shows that, in the stationary state, this rate of change in the entropy dissipated by the environment corresponds precisely to the entropy production rate according to the Lebowitz–Spohn formula, i.e., we precisely have

(59) $$\begin{array}{c} \sigma ={\dot{S}}_{\mathrm{tot}}={\dot{S}}_{\mathrm{res}} \end{array}$$

In order to show this, we need to explicitly write the expression of P[η], which in the stationary state is Gaussian:

(60) $$\begin{array}{c} P\left[\eta \right]\propto \exp\left\{-\frac{1}{2}\int dxdy\;\eta \left(x\right)c^{-1}\left(x,y\right)\eta \left(y\right)\right\}\;, \end{array}$$

where the kernel is the inverse of the stationary field correlator c(x,y)=〈η(x)η(y)〉. Then, by plugging this stationary probability into the expression of the current, one gets

(61) $$\begin{array}{cc} J[\eta ](x) & =P\left[\eta \right]\int dy\left[\lambda \left(x,y\right)\eta \left(y\right)+\frac{1}{2}\int dz\gamma \left(x,y\right)c^{-1}\left(y,z\right)\eta \left(z\right)\right]\;. \end{array}$$

Finally, by inserting the expression of the current Equation (61) into the expression of the reservoir entropy derivative ${\dot{S}}_{\mathrm{res}}\left(t\right)$, and by exploiting the same operator identities in Section 4, one gets

(62) $$\begin{array}{cc} {\dot{S}}_{\mathrm{res}} & =2\int dxdzdz^{\prime }dy\;[\lambda \left(x,y\right)\gamma^{-1}\left(x,z\right)\lambda \left(z,z^{\prime }\right)\langle \eta \left(z^{\prime }\right)\eta \left(y\right)\rangle \\ \; & +\frac{1}{2}\int dq\gamma \left(x,y\right)c^{-1}\left(y,q\right)\gamma^{-1}\left(x,z\right)\lambda \left(z,z^{\prime }\right)\langle \eta \left(z^{\prime }\right)\eta \left(q\right)\rangle )]\\ \; & =\mathrm{Tr}\left[2\Lambda^{T}\circ \Gamma^{-1}\circ \Lambda \circ C+\Lambda \right]\\ \; & =\mathrm{Tr}\left[\Lambda^{T}\circ \Gamma^{-1}\circ \Lambda \circ C\right]+\mathrm{Tr}\left[-\Lambda^{T}\circ \Gamma^{-1}\circ \left(C\circ \Lambda^{T}+\Gamma \right)+\Lambda \right]\\ \; & =\mathrm{Tr}\left[(\Lambda^{T}\circ \Gamma^{-1}-\Gamma^{-1}\circ \Lambda )\circ \Lambda \circ C\right]\;. \end{array}$$

The above formula coincides exactly with Equation (38). We have therefore shown an alternative way to derive the same expression obtained from the Lebowitz–Spohn approach.

## 6. Entropy Production in Bulk: An Explicit Formula

In order to provide more explicit expressions of the entropy production rate, a convenient and still quite general assumption, which simplifies a lot of the calculation, allowing for the final explicit expression, involves claiming translational invariance in space for the synaptic weight function. The assumption of translational invariance clearly does not fit the brain, if we think about it as a whole, since it does not have infinite extension, and it is not a periodic system. It has well-defined boundaries and a specific geometric shape. Nevertheless, if we imagine focusing on the properties of a small piece in the bulk of the brain, then, as is customary in the statistical mechanics approach to condensed matter, it makes sense to assume periodic boundary conditions and, as a consequence of that, translational invariance. In practice, this corresponds to the assumption that the synaptic weight kernel depends only on the difference between the two coordinates:

(63) $$\begin{array}{c} w(x,y)=w(x-y) \end{array}$$

By exploiting the translational invariance of the synaptic weight function, it is possible to consider the Fourier transform of the Langevin equation for the field fluctuations, Equation (17), thus resulting in *M* uncoupled equations, where *M* is the number of Fourier modes, $k\in [k_{0},\ldots ,k_{M}]$, of the following kind:

(64) $$\frac{\partial }{\partial t}\eta_{k}\left(t\right)=\lambda_{k}\;\eta_{k}\left(t\right)+\xi_{k}\left(t\right)\;$$

where

(65) $$\lambda_{k}=-\frac{1}{\tau }+\frac{f^{\prime }\left[u_{0}\right]}{\tau }\;w_{k}\;$$

where we introduced the Fourier transform of the synaptic weight

(66) $$\begin{array}{c} w_{k}=\frac{1}{{\left(2\pi \right)}^{d/2}}\int dx\;e^{ikx}\;w\left(x\right). \end{array}$$

At variance with the synaptic kernel w(x,y), the assumption of translational invariance for the noise kernel is quite natural; namely, we have γ(x,y)=γ(x−y). Since the noise correlator is then, by definition, symmetric, it follows that its Fourier transform $\gamma_{k}$ is a real function, i.e., γk=Re[γk]. Let us now specify, given the forward trajectory $\eta_{k}\left(s\right)$ with s∈[0,t], the definition of its corresponding backward trajectory $\bar{\eta_{k}\left(s\right)}$, which, consistent with its definition in real space, is defined as follows:

(67) $$\begin{array}{cc} \bar{\eta_{k}\left(s\right)} & =\eta_{k}\left(t-s\right), \end{array}$$

and also

(68) $$\begin{array}{cc} \frac{d\bar{\eta_{k}\left(s\right)}}{ds} & =-\frac{d}{du}\eta_{k}{|}_{u=(t-s)}. \end{array}$$

From the above definition, we therefore have that if the forward trajectory is a solution of the Langevin equation in Equation (64), the backward trajectory is then a solution of

(69) $$\begin{array}{c} \frac{d}{du}\eta_{k}{|}_{u=(t-s)}=-\lambda_{k}\;\eta_{k}\left(t-s\right)-\xi_{k}\left(t-s\right) \end{array}$$

In terms of the Fourier modes of the field, the probabilities of forward and backward trajectories can be rewritten, using a compact notation for time derivatives, ${\dot{\eta }}_{k}=\frac{d}{du}\eta_{k}$, as follows:

(70) $$\begin{array}{cc} P[\Omega_{0}^{t}] & \propto \exp\left\{-\frac{1}{2}\int_{0}^{t}ds\sum_{k}\;\left[{\dot{\eta }}_{k}^{*}\left(s\right)-\lambda_{k}^{*}\eta_{k}^{*}\left(s\right)\right]\gamma_{k}^{-1}\left[{\dot{\eta }}_{k}\left(s\right)-\lambda_{k}\eta_{k}\left(s\right)\right]\right\}, \end{array}$$

(71) $$\begin{array}{cc} P[{\bar{\Omega }}_{0}^{t}] & \propto \exp\left\{-\frac{1}{2}\int_{0}^{t}ds\sum_{k}\;\left[{\dot{\eta }}_{k}^{*}\left(t-s\right)+\lambda_{k}^{*}\eta_{k}^{*}\left(t-s\right)\right]\gamma_{k}^{-1}\left[\dot{\eta }\left(k,t-s\right)+\lambda_{k}\eta_{k}\left(t-s\right)\right]\right\}, \end{array}$$

which, exploiting the mathematical identity

(72) $$\begin{array}{cc} \int_{0}^{t}ds\;f\left(t-s\right) & =\int_{0}^{t}ds\;f\left(s\right), \end{array}$$

can be rewritten as

(73) $$\begin{array}{cc} P[\Omega_{0}^{t}] & \propto \exp\left\{-\frac{1}{2}\int_{0}^{t}ds\sum_{k}\;\frac{|{\dot{\eta }}_{k}{\left(s\right)|}^{2}+{\left|\lambda_{k}\eta_{k}\left(s\right)\right|}^{2}-{\dot{\eta }}_{k}^{*}\left(s\right)\lambda_{k}\eta_{k}\left(s\right)-{\dot{\eta }}_{k}\left(s\right)\lambda_{k}^{*}\eta_{k}^{*}\left(s\right)}{\gamma_{k}}\right\}, \end{array}$$

(74) $$\begin{array}{cc} P[{\bar{\Omega }}_{0}^{t}] & \propto \exp\left\{-\frac{1}{2}\int_{0}^{t}ds\sum_{k}\;\frac{|{\dot{\eta }}_{k}{\left(s\right)|}^{2}+{\left|\lambda_{k}\eta_{k}\right|}^{2}\left(s\right)+{\dot{\eta }}_{k}^{*}\left(s\right)\lambda_{k}\eta_{k}\left(s\right)+{\dot{\eta }}_{k}\left(s\right)\lambda_{k}^{*}\eta_{k}^{*}\left(s\right)}{\gamma_{k}}\right\}. \end{array}$$

Let us notice that in the above Equation (74), the field $\eta_{k}\left(s\right)$ and $\lambda_{k}$ take values in C, since there is no general reason to assume that w(x) has definite parity symmetry. Then, according to its definition, the entropy production rate for the stochastic Amari model with translational invariant synaptic weight is

(75) σ=limt→∞2t∫0tds∑kReλkη˙k*(s)ηk(s)γk=λkγk〈η˙k*ηk〉+λk*γk〈η˙kηk*〉

Further progress in the explicit calculation of the entropy production rate per Fourier mode $\sigma_{k}$ can be made by explicitly considering the formal solution of the Langevin Equation (64):

(76) $$\begin{array}{c} \eta_{k}\left(t\right)=e^{\lambda_{k}t}\;\eta_{k}\left(0\right)+\int_{0}^{t}ds\;e^{\lambda_{k}\left(t-s\right)}\;\xi_{k}\left(s\right), \end{array}$$

from which, under the not too restrictive assumption $\eta_{k}\left(0\right)=0$, one immediately gets

(77) $$\begin{array}{cc} \eta_{k}\left(t\right) & =\int_{0}^{t}ds\;e^{\lambda_{k}\left(t-s\right)}\;\xi_{k}\left(s\right) \end{array}$$

(78) $$\begin{array}{cc} {\dot{\eta }}_{k}\left(t\right) & =\xi_{k}\left(t\right)+\lambda_{k}\int_{0}^{t}ds\;e^{\lambda_{k}\left(t-s\right)}\xi_{k}\left(s\right) \end{array}$$

From the above expressions, we can compute, for instance, $\langle \dot{\hat{\eta }}\left(k,t\right){\hat{\eta }}^{*}\left(k,t\right)\rangle$:

(79) 〈η˙k(t)ηk*(t)〉=∫0tds˜eλk*(t−s˜)〈ξk(t)ξ^k*(s˜)〉+λk∫0tds˜dseλk*(t−s˜)+λk(t−s)〈ξk(s)ξk*(s˜)〉=γk12+λk∫0tdse[λk*+λk](t−s)=γk12+λkλk*+λke2Re[λk]t−λkλk*+λk.

Since we are interested in the value of the above correlation function at stationarity, we send t→∞, and assuming Re[λk]<0, we have

(80) 〈η˙kηk*〉=limt→∞〈η˙k(t)ηk*(t)〉=γk2Re[λk]λk*−λk2.

Correspondingly, it is straightforward to see that the complex conjugate of the same quantity is as follows:

(81) 〈η˙k*ηk〉=limt→∞〈η˙k*(t)ηk(t)〉=γk2Re[λk]λk−λk*2.

By plugging the above results on stationary correlators into the expression of entropy production in Equation (75), one gets the following:

(82) σk=λkγk〈η˙k*ηk〉+λk*γk〈η˙kηk*〉=iIm[λk](λk−λk*)2Re[λk]

and consequently

(83) σk=−Im2[λk]Re[λk].

This leads us to the final result for entropy production

(84) σ=−∑kIm2[λk]Re[λk],

which is a remarkably simple formula in terms of the eigenvalues $\lambda_{k}$ of the linearized Amari equation in Fourier space.

## 7. Conclusions

In this work, we derived an explicit and compact expression for the entropy production rate σ in the stochastic, linearized Amari neural field model, thereby providing a rigorous bridge between non-equilibrium statistical mechanics and large-scale neural dynamics. By computing σ, we showed that the emergence of irreversibility in the stochastic Amari model is entirely determined by the interplay between the symmetry of the synaptic kernel w(x,y) and the structure of the noise covariance γ(x,y). Moreover, we discussed the relationship between the entropy production rate σ, as defined by Lebowitz and Spohn, and the temporal variation in the system entropy ${\dot{S}}_{\mathrm{sys}}$, showing that, in a non-equilibrium stationary state, σ equals the entropy dissipated into the reservoir ${\dot{S}}_{\mathrm{res}}$. While this equivalence is well known in stochastic thermodynamics [27,29,41], we derived it by employing the functional formalism typical of neural field models. Thus, our derivation systematically connects the functional formulations of stochastic neural field theory [2,33] with the language of modern stochastic thermodynamics [29]. Most previous literature on the subject has aimed at developing methods for estimating the entropy production rate or other measures of irreversibility from experimental signals and, therefore, usually deals with finite-dimensional models [10,13,19,22]. In contrast, we carefully examined the mechanism of time reversal symmetry breaking in the idealized framework of the stochastic Amari model. It is worth emphasizing that this model lacks several biological ingredients, such as population heterogeneity, delays in signal propagation, and synaptic plasticity, since it describes a single scalar field η(x,t) and employs a time-independent coupling kernel w(x,y). Moreover, it is a phenomenological model, meaning that it is not derived from a microscopic description through a large-scale expansion. As explained in Section 3, fluctuations are externally imposed on the deterministic dynamics. To discuss, in the simplest way, the interplay between synaptic coupling and noise correlations, while avoiding certain interpretational subtleties, we focused on the case of additive Gaussian noise. Within this approximation, we are neglecting many possible sources of irreversibility such as those produced by non-Gaussian, non-Markovian, or multiplicative fluctuations. Despite these limitations, our analysis clarifies the conditions that w(x,y) and γ(x,y) must satisfy for linear, spatially extended neural fields to not produce entropy during evolution. For instance, the analysis in Fourier space, when translational invariance was assumed, revealed that the spatial correlations of fluctuations do not contribute to the breaking of time reversal symmetry, which originate solely from asymmetries in the coupling kernel. In this case, we also showed that the entropy production rate σ can be decomposed into positive contributions $\sigma_{k}$, one for each mode k. This decomposition, similar to that proposed by [23] but applied in wave-vector space k rather than in the frequency domain, provides a simple theoretical tool to probe dissipation in neuronal systems across different spatial scales associated with the Fourier modes. In conclusion, the simplified setting adopted here should be understood as a testbed for uncovering the mechanisms by which asymmetries in synaptic couplings and noise spatial correlations generate irreversible steady dynamics. Isolating these contributions to entropy production may prove useful when more realistic situations are considered. Moreover, it allowed us to establish the theoretical foundation for linking stochastic thermodynamics to spatially extended neural field models under minimal assumptions and can serve as a starting point for future explorations of energy dissipation and information flow in neuronal systems.

## Data Availability

The original contributions presented in this study are included in the article. Further inquiries can be directed to the corresponding author.
